# Parent-Child Assessment of Strengths and Difficulties of German Children and Adolescents Born With Esophageal Atresia

**DOI:** 10.3389/fped.2021.723410

**Published:** 2021-09-17

**Authors:** Stefanie Witt, Jens Dingemann, Michaela Dellenmark-Blom, Julia Quitmann

**Affiliations:** ^1^Department of Medical Psychology, Center for Psychosocial Medicine, University Medical Center Hamburg-Eppendorf, Hamburg, Germany; ^2^Center of Pediatric Surgery, Hannover Medical School and Bult Children's Hospital, Hannover, Germany; ^3^Department of Pediatrics, Institute of Clinical Sciences, Queen Silvia Children's Hospital, Gothenburg, Sweden; ^4^Department of Pediatric Surgery, Queen Silvia Children's Hospital, Gothenburg, Sweden

**Keywords:** internalizing and behavioral problems, pediatric patients, esophageal atresia (EA), rare disease (RD), parent-child perspective

## Abstract

**Introduction/Aim:** Children and adolescents with a chronic somatic disease have a higher risk of developing psychological disorders than healthy peers. Therefore, we aim to investigate internalizing and behavioral problems in pediatric patients with esophageal atresia (EA) and compare this sample with German reference values using both childrens' self-reports and parents' proxy reports.

**Methods:** The present cross-sectional study is part of the German-Swedish EA-QOL study developing a condition-specific instrument to assess Health-related Quality of Life in children and adolescents born with EA from both self and proxy perspectives. The current analyses use data from the German sample collected within the field test phase. Participants were enrolled from the Medical School Hannover and “Auf der Bult” Children's Hospital, Hannover. The cooperating clinicians provided the medical records while socio-demographic information was collected through the parent-report within the questionnaires. We used the *Strengths and Difficulties Questionnaire (SDQ)* to measure internalizing and behavioral problems of children and adolescents born with EA ranging from 2 to 18 years.

**Results:** A total of 51 families participated in the field test phase. Eighty-eight parent reports and 22 child reports were included in the analyses. While the parents' perspective from the SDQ leads to a higher percentage of abnormal or borderline behavior, there is no difference to the reference group from the children's perspective.

**Conclusion:** Incorporating routine psychological assessment into pediatric health care can help improve understanding of the burden of illness, examine treatment outcomes, assess the quality of care, and tailor interventions to meet patient and parent needs. Involving the whole family can help develop appropriate and functional coping strategies. From our point of view, it is necessary to address parental needs and concerns as well in order to provide the best possible holistic development in the family system. The family is the basis for the children's successful development, especially for children with special health care needs.

## Introduction

The progress in medicine, surgery, and research has led to an increased survival rate for patients affected by a rare disease, such as patients with esophageal atresia (EA). Esophageal atresia describes a congenital malformation characterized by a discontinuity of the esophagus (1). In the EU, EA's prevalence rate with or without tracheoesophageal fistula was 2.36 per 10,000 live births from 2011 to 2017 (2). EA has several anatomic subtypes, varying with the tracheoesophageal fistula's presence and location and the distance between the upper and lower esophageal pouches (3, 4).

When classified in 1929, the survival rate of the disease was around 0%. Due to the development of new treatment methods (5), the children's survival rate increased from 40% in 1,941 to nowadays 90–95% (6, 7), enabling the children to participate in a normal lifestyle. However, studies indicate that these children are affected by the disease in their later life by suffering from high morbidity (8). EA patients report common gastroesophageal sequelae of their malformation, such as swallowing difficulties and esophageal dysmotility leading to impaction of food in the esophagus (also known as acute “bolus obstruction”). The presence of two or more malformations in EA patients is called Vacterl association (Vertebral, Anorectal, Cardiac, Tracheo-Esophageal. Renal and Limb malformations). After repair of EA, 43–70% of children have been observed to have dysphagia (9, 10). Up to 71% have developed anastomotic strictures with a need for esophageal dilation (9). Moreover, 44–65% of the children may suffer from gastrointestinal reflux disease (9, 11). The latter can lead to cough, heartburn, and vomitus (12). It may also induce or aggravate esophageal stenosis requiring endoscopic dilatations or even re-do surgery.

Prior studies have already stressed that children with EA may be at risk of developing mental health and psychosocial problems: In 1990, a study on children with EA described tendencies of contact behavior disorders, separation anxiety, and regressive behavior. Lehner (13) explains this behavior with the numerous hospital stays, separation from the mother, and painful surgical interventions. Developmental delay was reported in 25% of EA patients at school-age. Another study showed a significantly higher proportion of EA children with problematic behavior compared to population-based references (14). In a study by Ludman and Spitz (15), most school-aged EA patients needed special-need units at school or additional support. Finally, longitudinal studies reported that 31% of EA infants showed mental disorders at the age of 1 year and identified post-traumatic symptoms of the mother, more than one surgical intervention, and moderate or severe family strain to predict mental disorders of the infant (16).

Since a recent study from Mikkelsen et al. (17) stressed the significant burden of adolescent patients lasting several years after EA surgery and hospital stay, this current study focuses on internalizing and behavioral problems in pediatric patients with EA. It compares this sample with German reference values using both children's self-reports and parents' proxy reports. Additionally, to get a comprehensive view of the affected children and their psychosocial situation, we aim to examine parent-child agreement and predictors of internalizing and behavioral problems. From former studies investigating the health-related quality of life (HrQoL) in children and adolescents with chronic health conditions, we know that parents tend to underrate generic HrQoL of the children (18–22) while children tend to report more problems than their parents (23). This limited parent-child agreement calls for capturing both patients' and parents' reports to understand better the impact of EA on children's psychosocial situation.

## Methods

### Study Design

The present study is part of the German-Swedish EA-QOL study developing a condition-specific instrument to assess HrQoL in children and adolescents born with EA from self and proxy perspectives (24). The current analysis uses data from the German sample collected within the field test phase.

Participants from Hannover Medical School (MHH) and “Auf der Bult” Children's Hospital, Hannover were enrolled if the pediatric patients met the following inclusion criteria: (a) clinical diagnosis of EA, (b) children aged 2–18 years, (c) absence of other severe health conditions which did not include conditions with associations with EA such as tracheomalacia or tracheostomy, and (d) sufficient German language proficiency. Eligible participants were identified by the pediatric surgeons based on the children's medical records.

Parents and children aged eight and older received detailed information about the study. Informed consent was obtained from the parents and informal assents from the children and adolescents aged 8 years and older. Questionnaires for children aged 8–18 years and parents of children aged 2–18 years were sent out between September and November 2016, including detailed information about the study, consent forms, and a pre-stamped envelope. Families received a maximum of three reminders to increase response rates. The data were anonymized and entered into SPSS for data analysis. The consent form and questionnaires will be stored separately for 10 years in the Department of Medical Psychology, University Medical Center Hamburg-Eppendorf.

Ethical approval was obtained before the commencement of the study (2396-2015 Ethical review board of the MHH).

### Measurements

The cooperating clinics MHH and “Auf der Bult” provided medical records for pediatric patients such as birth weight, diagnosis, comorbidities, type, and surgery date. Socio-demographic information was collected through the parent report within the questionnaires.

We used the *Strengths and Difficulties Questionnaire (SDQ)* (25) to measure children's internalizing and behavioral problems. The *SDQ* is a validated instrument to screen for mental health disorders. It is available as parent reports (and proxy reports for teachers) and as child reports. (26). The German version of the SDQ is validated by Woerner et al. (27) and Klasen et al. (28), the internal consistency (Cronbach's Alpha) is 0.82.

The SDQ contains 25 items, which form five scales. A *total difficulties score* can be calculated using the four scales of *emotional symptoms, conduct problems, hyperactivity/inattention*, and *peer relationship problems;* higher scores indicate more problems. The subscale *prosocial behavior* is inverted; higher scores represent more prosocial behavior.

Cut-off values for classification into *normal, borderline*, or *abnormal* are used to simplify the interpretation (27). According to the standardization, 80% of the children should be classified as *normal*, 10% as *borderline*, and 10% as *abnormal* (28).

Additionally, we calculated German reference values for child self-reports and parent proxy-reports from the “German Health Interview and Examination Survey for Children and Adolescents” (KIGGS) using a Public User File (PUF) (29). Hence, our defined study sample (parents of children aged 2–7 years, parents of children aged 8–18 years, and children aged 8–18 years) was compared to the population-based results of the age groups of 2–7 and 8–17 years.

### Data Analysis

Data were analyzed using Statistical Package for the Social Sciences (SPSS) Version 25 (30). The subscales of the SDQ emotional symptoms, conduct problems, hyperactivity/inattention, peer relationship, prosocial behavior, and the total difficulties score were calculated. Mean values (M) and standard deviations (SD) were calculated for continuous variables. Frequencies and percentages were calculated for categorical variables.

We investigated differences in the dichotomy SDQ classifications (normal vs. borderline/abnormal) regarding EA patients with or without associated anomalies and used chi-square tests. To investigate mean differences in the subscales and the total score of our sample of EA patients and their parents and a population-based reference sample, we performed Student's *t*-tests. We investigated parent-child-agreement using intraclass correlation (ICCs) [two-way mixed model, absolute agreement, 95% confidence interval (CI)]. ICCs were interpreted according to Koo and Lee (31). ICCs < 0.50 will be interpreted as poor agreement, ICCs between 0.50 and 0.75 as moderate agreement, ICCs between 0.75 and 0.90 as good agreement, and ICCs higher than 0.90 will be interpreted as excellent agreement. We performed multiple regression analyses to examine predictors of the SDQ total score from child- and parent perspectives, including socio-demographic variables of the pediatric patients (age, gender) and their parents (educational level) as well as clinical factors (associated anomalies, severity level). The significance level was *p* ≤ 0.05.

## Results

### Demographics

A total of 51 families participated in the field test phase. The analysis included 88 parent reports (from mothers and fathers) of pediatric patients aged 2–18 years and 22 child reports of children and adolescents aged 8–18. The sample characteristics of the pediatric patients and their parents are presented in Table 1.

**Table 1:** Sample characteristics.

**Child characteristics**	**Children 2–7 (** ***n*** **=** **27)**				**Children 8–18 (** ***n*** **=** **24)**			
	***M***	**SD**	**Min**	**Max**	***M***	**SD**	**Min**	**Max**
Age	4.9	1.6	2.3	7.4	12.7	2.9	8.4	18.2
Gender	***n***	**%**			***n***	**%**		
Male	14	51.9			14	58.3		
Additional support in school	8^1^	36.4			5^2^	21.7		
Primary esophageal repair	24	88.9			22	91.7		
Revisional surgery	3	11.1			3	12.5		
Associated anomalies	13	48.1			15	62.5		
Cardiovascular	7	25.9			7	29.2		
Gastrointestinal (excluding anorectum)	2	7.4			2	8.3		
Anorectal	3	11.1			5	20.8		
Uro-genital	5	18.5			1	4.2		
Limb	3	11.1			1	4.2		
Vertebral-skeletal	2	7.4			1	4.2		
Central nervous system	3	11.1			2	8.3		
Respiratory	4	14.8			2	8.3		
Eye	0	0.0			1	4.2		
Ear	3	11.1			3	12.5		
Vacterl	5	18.5			1	4.2		
Classification of EA								
Gross A	4	14.8			2	8.3		
Gross B	0	0.0			1	4.2		
Gross C	22	81.5			21	87.5		
Gross D	1	3.7			0	0.0		
Number of associated anomalies	1.5	2.0	0	6	1.2	1.3	0	4
Severity level^b^								
Mild-moderate	14	51.9			15	62.5		
Severe	13	48.1			9	37.5		
**Parent characteristics**	**Parents of children 2-7 (** ***n*** **=** **49)**				**Parents of children 8-18 (** ***n*** **=** **39)**			
	***M***	**SD**	**Min**	**Max**	***M***	**SD**	**Min**	**Max**
Age	38.4	7.0	24	55	46.9	7.1	30	63
	***n***	**%**			***n***	**%**		
Mother	25	51			22	56.4		
Educational level^a^								
Low	3	6.1			4	10.3		
Medium	15	60.6			15	38.5		
High	3	6.1			20	51.2		

*n, number; M, Mean; SD, Standard deviation; min, Minimum; max, Maximum; Vacterl, Vertebral, Anorectal, Cardiac, Tracheo-Esophageal. Renal and Limb malformations*.

1 *5 missing values*.

2 *1 missing value*.

a *Classification of educational level: low – no degree, elementary school; medium – MSA; high – abitur/University degree*.

b *The severity of EA was divided into mild/moderate and severe according to predefined clinical criteria published elsewhere (32)*.

### SDQ-Classification of EA Patients

The classification of the SDQ scales shows that parents of young patients aged 2–7 years reported more internalizing and externalizing problems than parents of children and adolescents aged 8–18 years. The percentages for scores categorized as borderline or abnormal are higher in young children's parents than in parents of adolescents born with EA. Forty-eight percentage of the mothers, respectively, 41.6% of the fathers of young EA patients report their children's behavior as classified as borderline or abnormal for the total difficulties score. At the same time, this is true for 20.8% of the parents of adolescents born with EA. No substantial differences between parents of both age groups are found for prosocial behavior. Eighty-eight percentage of the mothers, respectively, 79.2% of the fathers of young report prosocial behavior in the normal range compared to 100% of mothers, respectively, 88.2% of fathers of adolescent patients. The assessment of mothers and fathers shows only small differences in total. The highest differences we found for the classification of abnormal behavior for the scales hyperactivity/inattention (22.7 vs. 5.9%; 8–18 years), emotional symptoms (28 vs. 12.5%; 2–7 years), and the total difficulties score (36 vs. 54.2%; 2–7 years) with mothers reporting more problems. Fathers reported more problems than mothers for normal behavior in the scale emotional symptoms (60 vs. 79.2%, 2–7 years), the borderline classification of the scale hyperactivity (0 vs. 17.6%, 8–18 years), and the abnormal classification for the scale conduct problems (28 vs. 41.7%, 2–7 years) (Table 2).

**Table 2:** Classification of the Strengths and difficulties questionnaire.

**SDQ-Classification^a^**		**Parents of children aged 2–7 years**				**Parents of children aged 8–18 years**				**References^b^**	**Children aged 8–18 years**	
		**Mothers** ***n*** **=** **25**		**Fathers** ***n*** **=** **24**		**Mothers** ***n*** **=** **22**		**Fathers** ***n*** **=** **17**			**Children** ***n*** **=** **21**	
		***n***	**(%)**	***n***	**(%)**	***n***	**(%)**	***n***	**(%)**	**(%)**	***n***	**(%)**
Total difficulties score	Normal	13	52	14	58.3	17	77.3	15	88.2	81.6	20	95.2
	Borderline	3	12	5	20.8	4	18.2	1	5.9	8.4	0	0
	Abnormal	9	36	5	20.8	1	4.5	1	5.9	10.0	1	4.8
Emotional symptoms	Normal	15	60	19	79.2	20	90.9	15	88.2	86.0	19	90.5
	Borderline	3	12	2	8.3	1	4.5	2	11.8	6.3	1	4.8
	Abnormal	7	28	3	12.5	1	4.5	0	0	7.7	1	4.8
Conduct problems	Normal	14	56	12	50	17	77.3	14	82.4	84.7	18	85.7
	Borderline	4	16	2	8.3	2	9.1	2	11.8	8.7	2	9.5
	Abnormal	7	28	10	41.7	3	13.6	1	5.9	6.6	1	4.8
Hyperactivity/inattention	Normal	18	72	16	66.7	17	77.3	13	76.5	85.3	13	61.9
	Borderline	2	8	2	8.3	0	0	3	17.6	4.9	3	14.3
	Abnormal	5	20	6	25	5	22.7	1	5.9	9.8	5	23.8
Peer relationship	Normal	16	64	13	54.2	19	86.4	14	82.4	86.7	19	90.5
	Borderline	1	4	2	8.3	0	0	1	5.9	6.3	1	4.8
	Abnormal	8	32	9	37.5	3	13.6	2	11.8	7.0	1	4.8
Prosocial behavior	Normal	22	88	19	79.2	22	100	15	88.2	84.4	20	95.2
	Borderline	2	8	2	8.3	0	0	1	5.9	8.5	0	0
	Abnormal	1	4	3	12.5	0	0	1	5.9	7.1	1	4.8

*n, number*.

a *Klasen et al. (28)*.

b *Woerner et al. (27)*.

In comparison to the distribution within a German reference population of children aged 6–16 years, our results reveal that especially parents of young EA patients (2–7 years) report more difficulties except for the scale prosocial behavior and father-reported emotional symptoms. In contrast, the percentages of the classifications for parents of adolescents born with EA compared to the reference population show small differences in normal, borderline, and abnormal behavior (Table 2).

The child reports of EA patients aged 8–18 years show a higher percentage of children in the normal classification of the total difficulties score (95.2%) than their parents (varies between 52 and 88.5%). A higher rate of adolescents reported SDQ scores categorized as borderline (14.3%) or abnormal (23.8%) in the scale hyperactivity/inattention in comparison to their mothers (0% borderline and 22.7% abnormal) and their fathers (17.6% borderline and 5.9% abnormal) (Table 2).

### Comparison of Patients With or Without Associated Anomalies

Parents of EA patients aged 2–7 years whose child showed the presence of at least one associated anomaly reported more often a total difficulties score within the classification borderline/abnormal (chi^2^ = 5.89, *p* = 0.02) and peer relationship (chi^2^ = 13.01, *p* ≤ 0.01). Parents of children aged 8–18 years with at least one associated anomaly reported more often borderline/abnormal behavior reading hyperactivity and inattention (chi^2^ = 4.39, *p* = 0.04) than parents of children without such an anomaly. We did not found any significant differences analyzing the self-report of EA patients aged 8–18 years (Table 3).

**Table 3:** Comparison of SDQ-classifications for EA patients with an without associated anomalies.

**Parents of children aged 2–7 years (PR;** ***n*** **=** **49)**		**Associated anomalies**			
		**Yes**	**No**	**Chi^2^**	***p***
Total difficulties score	Normal	9	18	5.89	**0.02**
	Borderline/abnormal	17	7		
Emotional symptoms	Normal	16	18	0.16	0.69
	Borderline/abnormal	8	7		
Conduct problems	Normal	12	14	0.18	0.67
	Borderline/abnormal	12	11		
Hyperactivity/inattention	Normal	14	20	2.71	0.10
	Borderline/abnormal	10	5		
Peer relationship	Normal	8	21	13.01	≤ 0.01
	Borderline/abnormal	16	4		
Prosocial behavior	Normal	18	23	2.59	0.11
	Borderline/abnormal	6	2		
**Parents of children aged 8–18 years (PR;** ***n*** **=** **42)**		**Associated anomalies**			
		**Yes**	**No**	**Chi** ^2^	***p***
Total difficulties score	Normal	18	15	3.54	0.06
	Borderline/abnormal	8	1		
Emotional symptoms	Normal	21	15	1.36	0.24
	Borderline/abnormal	5	1		
Conduct problems	Normal	18	14	1.82	0.18
	Borderline/abnormal	8	2		
Hyperactivity/inattention	Normal	17	15	4.39	0.04
	Borderline/abnormal	9	1		
Peer relationship	Normal	19	15	2.75	0.10
	Borderline/abnormal	7	1		
Prosocial behavior	Normal	24	18	1.29	0.26
	Borderline/abnormal	2	0		
**Children aged 8–18 years (SR;** ***n*** **=** **22)**		**Associated anomalies**			
		**Yes**	**No**	**Chi** ^2^	***p***
Total difficulties score	Normal	13	8	0.60	0.44
	Borderline/abnormal	1	0		
Emotional symptoms	Normal	12	8	1.26	0.26
	Borderline/abnormal	2	0		
Conduct problems	Normal	11	8	1.96	0.16
	Borderline/abnormal	3	0		
Hyperactivity/inattention	Normal	7	7	3.09	0.08
	Borderline/abnormal	7	1		
Peer relationship	Normal	11	8	1.96	0.16
	Borderline/abnormal	3	0		
Prosocial behavior	Normal	13	8	0.60	0.44
	Borderline/abnormal	1	0		

*PR, proxy-report; SR, self-report; n, number; p, two-tailed significance*.

### Comparison With Population-Based Reference Values

Mothers of pediatric patients born with EA aged 2–7 years reported significantly higher scores in the scales emotional symptoms [*t*_(24)_ = 2.42, *p* = 0.02], peer relationship [*t*_(24)_ = 2.14, *p* = 0.04], and total difficulties score [*t*_(24)_ = 2.12, *p* = 0.05] in comparison to the population-based reference group. Fathers of children aged 2–7 years reported significantly higher scores in the scales peer relationship [*t*_(23)_ = 3.20, *p* ≤ 0.01] as well as the total difficulties score [*t*_(23)_ = 2.67, *p* = 0.01]. Mothers of children and adolescents aged 8–18 years reported significantly higher scores in the scale hyperactivity/inattention [*t*_(21)_ = 2.26, *p* = 0.04] than the reference group. Neither fathers of children and adolescents aged 8–18 years nor the pediatric patients themselves reported significant differences (Table 4).

**Table 4:** Comparison of SDQ-values for EA patients with Germen reference values.

	**Mothers of children born with EA aged 2–7 years (PR)**						**Fathers of children born with EA aged 2–7 years (PR)**						**Reference values^a^**		
	***n***	***M***	**SD**	***T***	**df**	***p***	***n***	***M***	**SD**	***T***	**df**	***p***	***n***	***M***	**SD**
Total difficulties score	25	11.92	8.67	2.12	24	0.05	24	11.67	6.26	2.67	23	**0.01**	3,145	8.25	4.56
Emotional symptoms	25	2.96	2.73	2.42	24	0.02	24	1.75	2.29	0.24	23	0.82	3,146	1.64	1.59
Conduct problems	25	2.52	2.45	0.53	24	0.60	24	3.04	2.39	1.61	23	0.12	3,145	2.26	1.52
Hyperactivity/inattention	25	4.16	3.00	1.56	24	0.13	24	4.42	2.93	2.00	23	0.06	3,146	3.22	2.18
Peer relationship	25	2.28	2.67	2.14	24	0.04	24	2.46	2.02	3.20	23	0.01	3,147	1.14	1.40
Prosocial behavior	25	7.72	1.84	−0.98	24	0.34	24	7.58	2.40	−1.02	23	0.32	3,146	8.08	1.52
	**Mothers of children born with EA aged 8–18 years (PR)**						**Fathers of children born with EA aged 8–18 years (PR)**						**Reference values^b^**		
	***n***	***M***	**SD**	***T***	**df**	***p***	***n***	***M***	**SD**	***T***	**df**	***p***	***n***	***M***	**SD**
Total difficulties score	22	8.32	5.57	0.20	21	0.84	17	7.65	5.01	−0.36	16	0.73	7,208	8.08	4.99
Emotional symptoms	22	1.41	1.53	−1.75	21	0.10	17	1.53	1.28	−1.45	16	0.17	7,213	1.98	1.88
Conduct problems	22	1.50	1.63	−1.15	21	0.26	17	1.29	1.21	−2.06	16	0.06	7,214	1.90	1.52
Hyperactivity/inattention	22	4.18	2.70	2.26	21	0.04	17	3.59	2.32	1.26	16	0.23	7,212	2.88	2.17
Peer relationship	22	1.23	1.72	−0.23	21	0.82	17	1.24	1.92	−0.16	16	0.88	7,211	1.31	1.53
Prosocial behavior	22	8.45	1.37	0.36	21	0.72	17	8.00	1.80	−0.80	16	0.44	7,212	8.35	1.56
	**Children born with EA aged 8–18 years (SR)**												**Reference values^c^**		
	***n***	***M***	**SD**	***T***	**df**	***p***							***n***	***M***	**SD**
Total difficulties score	22	10.01	5.58	0.65	21	0.52							4,952	9.32	4.42
Emotional symptoms	22	2.14	1.86	−0.54	21	0.60							4,952	2.35	1.89
Conduct problems	22	1.77	1.31	0.58	21	0.57							4,952	1.61	1.25
Hyperactivity/inattention	22	4.45	2.74	1.43	21	0.17							4,952	3.62	2.05
Peer relationship	22	1.73	2.07	−0.03	21	0.98							4,952	1.74	1.39
Prosocial behavior	22	8.36	1.53	0.38	21	0.71							4,952	8.24	1.48

*PR, proxy-report; SR, self-report; n, number; M, Mean; SD, Standard Deviation; T, t-value; df, degrees of freedom; p, two-tailed significance*.

a *Reference values of parents with children aged 2–7 years (PR) from the KIGGS Public User File (29)*.

b *Reference values of parents with children aged 8–17 years (PR) from the KIGGS Public User File (29)*.

c *Reference values of children with children aged 8–17 years (SR) from the KIGGS Public User File (29)*.

### Parent-Child Agreement

At the individual level, ICCs for parent-child agreement, ranged between 0.46 (prosocial behavior; mother-child-dyads) and 0.91 (total difficulties score; father-child-dyads). Mother-child agreement showed good agreement for the total difficulties score [ICC (CI) = 0.78 (0.50 to 0.91)], as well as the scales conduct problems [ICC (CI) = 0.87 (0.69 to 0.95)] and hyperactivity/inattention [ICC (CI) = 0.85 (0.64 to 0.94)]. The agreement of mother-child report on the scales emotional symptoms [ICC (CI) = 0.73 (0.36 to 0.89)] and peer relationship [ICC (CI) = 0.73 (0.35 to 0.89)] yielded moderate agreement, while mother-child agreement on the scale prosocial behavior [ICC (CI) = 0.46 (−0.36 to 0.79)] showed poor agreement.

ICCs for father-child agreement yielded excellent agreement on the total difficulties score [ICC (CI) = 0.91 (0.74 to 0.97)] as well as good agreement for the scales hyperactivity/inattention [ICC (CI) = 0.85 (0.59 to 0.95)], peer relationship [ICC (CI) = 0.85 (0.57 to 0.95)] and prosocial behavior [ICC (CI) = 0.81 (−0.47 to 0.93)]. The scales emotional symptoms [ICC (CI) = 0.67 (0.03 to 0.88)] and conduct problems [ICC (CI) = 0.60 (−0.14 to 0.86)] showed moderate agreement between father-child dyads (Table 5).

**Table 5:** Inter-rater reliability.

	**Dyads *n***	**Mother-report**		**Child-report**		**ICC (CI)**
		***M***	**SD**	***M***	**SD**	
Total difficulties score	21	8.14	0.56	9.86	5.61	0.78 (0.50 to 0.91)
Emotional symptoms	21	1.43	1.57	2.05	1.86	0.73 (0.36 to 0.89)
Conduct problems	21	1.47	1.66	1.76	1.34	0.87 (0.69 to 0.95)
Hyperactivity/inattention	21	4.19	2.77	4.57	2.75	0.85 (0.64 to 0.94)
Peer relationship	21	1.05	1.53	1.48	1.75	0.73 (0.35 to 0.89)
Prosocial behavior	21	8.57	1.29	8.43	1.54	0.46 (−0.36 to 0.79)
	**Dyads** ***n***	**Father-report**		**Child-report**		**ICC (CI)**
		***M***	**SD**	***M***	**SD**	
Total difficulties score	16	7.56	5.16	9.00	6.11	0.91 (0.74 to 0.97)
Emotional symptoms	16	1.56	1.31	1.81	1.83	0.67 (0.03 to 0.88)
Conduct problems	16	1.38	1.20	1.69	1.40	0.60 (−0.14 to 0.86)
Hyperactivity/inattention	16	3.56	2.39	4.25	2.86	0.85 (0.59 to 0.95)
Peer relationship	16	1.06	1.84	1.25	1.91	0.85 (0.57 to 0.95)
Prosocial behavior	16	8.00	1.86	8.31	1.62	0.81 (0.47 to 0.93)

*n, sample size; M, Mean; SD, standard deviation; ICC, Intraclass correlation coefficient; CI, Confidence Intervall; df, degrees of freedom; p, two-tailed significance*.

### Predictors of SDQ Total

The multivariate regression analyses demonstrated that our model explained a substantial variance of the parent-reported total difficulties score of the SDQ for children aged 2–7 years [*F*_(5, 43)_ = 3.29; *p* = 0.01]. The number of associated anomalies (β = 0.46; *p* ≤ 0.02) and parents' educational level (β = −0.29; *p* = 0.03) were identified to be significant predictors of the total difficulties score of young pediatric EA patients. A higher number of anomalies were associated with a higher total difficulties score in the parent report. Higher parental education was associated with a lower total difficulties score in patients aged 2–7 years (Table 5). Child's age (β = −0.32; *p* = 0.05) was identified to be a significant predictor for parent-reported problems in children and adolescents aged 8–18 years of age, while the model did not explain a substantial variance [*F*_(5, 33)_ = 2.23; *p* = 0.07]. The same independent variables did not contribute to explaining a significant variance for the child-reported total difficulties score of the SDQ [*F*_(5, 16)_ = 0.85; *p* = 0.53] (Table 6).

**Table 6:** Multivariate linear regression: predictors of SDQ total from both child-report and parent-report.

**Independent variables**	**SDQ total**								
	**2–7 years**			**8–18 years**					
	**Parent-reported (** ***n*** **=** **49)**			**Child-reported (** ***n*** **=** **22)**			**Parent-reported (** ***n*** **=** **39)**		
	**β**	***t***	***p***	**β**	***t***	***p***	**β**	***t***	***p***
Child's age	0.13	0.94	0.35	−0.35	−1.50	0.15	−0.32	−2.03	0.05
Child's gender^a^	−0.05	−0.35	0.73	0.18	0.78	0.45	0.11	0.71	0.48
Severity level^b^	−0.20	−1.29	0.20	−0.29	−0.89	0.39	−0.50	−0.31	0.76
Number of associated anomalies	0.46	2.87	≤ 0.01	0.25	0.89	0.39	0.31	1.95	0.06
Educational level^c^	−0.29	−2.21	0.03	0.22	0.83	0.42	−0.15	−0.94	0.35
Model summary	*R*^2^ = 0.28			*R*^2^ = 0.21			*R*^2^ = 0.28		
	*F*_(5, 43)_ = 3.29			*F*_(5, 16)_ = 0.85			*F*_(5, 43)_ = 3.29		
	*p* = 0.01			*p* = 0.53			*p* = 0.01		

a *Child's gender: 0 – female, 1 – male*.

b *Severity level: 0 – mild/moderate, 1 – severe*.

c *Educational level: 0 – low/middle (no degree, elementary school, junior high school), 1 – high (high school, college, university)*.

## Discussion

Since the reports on the psychosocial situation in pediatric EA patients are few and inconsistent, this study examined the internalizing and behavioral problems and prosocial behavior in children with EA from their own and their parent's perspectives. Our findings reveal different perceptions of internalizing and externalizing behavior of parents and pediatric patients born with EA. Results from HrQoL research support the different perceptions of parents and pediatric patients, showing that a comprehensive assessment involving various stakeholders, particularly the patients and their parents are necessary to allow a broader view on the whole family needs (21, 33, 34).

According to Mikkelsen et al. (17), we identified a greater proportion of internalizing and behavioral problems in German children born with EA than a German reference group. In our sample, this is particularly true for pediatric patients for EA patients aged 2–7 years using mothers' and fathers' reports. On the one hand, the adolescents aged 8–18 years from our sample did not report significantly more behavior problems than the German reference population, in contrast to the results of Mikkelsen et al. (17). On the other hand, our results are in line with the German BELLA presenting a higher SDQ score for parent-reports than self-reports (35). By the same time, pediatric patients born with EA did report high scores of generic HrQoL (36), and no statistically relevant differences were reported by EA patients using self-reports than healthy controls (37).

Thus, the higher percentage of EA patients reporting mental health problems regarding the total difficulties score can be confirmed for mothers and fathers reports of 2–7 years old EA patients in Germany. Higher scores for emotional problems reported by mothers can also be confirmed for the group of young EA patients (17) but not for father-reported difficulties.

Our results showed that associated anomalies are associated with a higher risk of borderline or abnormal behavior from the parent's perspective. Similar results were reported by Glinianaia et al. (38), summarizing that HrQoL of pediatric patients born with EA is affected by associated anomalies, and Peetsold et al. (39), reporting that associated anomalies negatively affect the general health perception of EA patients.

However, no significant associations were found for child-reported strengths and difficulties. Negative associations between the presence of associated anomalies and health-related quality of life were reported by Dellenmark-Blom et al. (40) for self-reported condition-specific HrQoL focusing on EA-specific aspects of daily life. Also, Peetsold et al. (39) confirm that these associations were only found for parent-reported HrQoL and not for child-reported outcomes. In contrast, no significant associations were found for generic HrQoL and the presence of Vacterl for both parent-reports and child reports (41).

According to the findings from Flieder et al. (41), we assume that older children and adolescents aged 8–18 years might have developed functional coping strategies to handle their health conditions, which obviously might result in an adapted and mostly inconspicuous social behavior (42, 43).

Accordingly, we identified the child's age as a significant predictor of parent-reported problem scores for EA adolescents aged 8 to 18. The younger the adolescent was, the higher the parent-reported total difficulties scores. By the same time, age did not predict the total difficulties score in parents of young EA patients aged 2–7 years. In line with current research, the patients' age (in our study: 2–7 years vs. 8–18 years) seems to be a predominant factor influencing emotional well-being and behavior. Also, HrQoL research in pediatric patients born with EA confirms a negative effect of age on general health perception (39).

While mental disorders were diagnosed in about one-third of 1-year-old EA patients (16), there were no differences regarding the mental health status of adolescent EA patients compared to healthy peers (44). The findings of Faugli et al. (44) and Faugli et al. (16) confirm the previously mentioned hypothesis of good adaptation and probably appropriate coping strategies and should be investigated further.

In contrast to results from the German validation study for the self-report form of the SDQ (45) reporting an association of the child's gender, we did not found gender to be a significant predictor for the total difficulties score.

We also found that more associated anomalies and low parental education levels predicted internalizing and behavior problems for children aged 2–7 years from the parent's perspective. Thus, our findings also strengthen the results from a study by Dingemann et al. (46), which describes low maternal education as a risk factor for additional congenital malformations and a higher incidence of post-operative complications in newborns with isolated congenital malformations. By the same time, none of the included variables predicted the total difficulties score for child-reported mental health.

Mother-child agreement in this study was poor to moderate, with children reporting higher scores for internalizing and externalizing behavior problems than their mothers and lower scores for prosocial behavior. Father and child reports showed moderate to excellent agreement, with children reporting higher problem scores and higher prosocial behavior scores. The agreement on emotional symptoms was only moderate for both mother-child dyads and father-child dyads. These results align with current research, reporting higher agreements for externalizing subscales than internalizing scales (47). Items and scales reflecting observable aspects of daily life tend to result in higher parent-child agreement than items that reflect emotional aspects or social relationships (48, 49). Parent-child agreement of emotional well-being and behavior was reported to be low to moderate in several studies across different age groups and states of health condition (48, 50–52). Different interpretations of the questions, lack of parental awareness of children's behavior, and different perceptions of thresholds are mentioned as possible reasons (48). Besides, the parental burden is associated with lower parent-child agreements (19) and should be considered by clinicians when assessing the psychosocial situation of children with EA.

## Limitations

Some limitations need to be acknowledged when interpreting the results and findings of the present study. Although EA is a rare chronic health condition with a prevalence of 2.36 per 10,000 live births (2), the small sample size does not allow the generalization of the findings. We used a non-probabilistic sampling strategy, which may influence the levels of parent-child agreement. We have to consider that parents who participated may be more involved in pediatric healthcare than parents who had denied participating. In contrast to many other studies, both mothers and fathers participated in our study.

Furthermore, the study's cross-sectional design, which precludes the inference of causality, should be considered when interpreting these results. We compared the SDQ score of the EA patients and their parents. Since we assessed internalizing and externalizing behavior using written questionnaires *via* paper and pencil, parents and children could fill out the questionnaire together, leading to bias. Another bias may result from our exclusion criteria. Children and adolescents with severe chronic comorbidity or lack of knowledge of the German language were excluded systematically, resulting in another selection Bias.

## Conclusion

Our study highlights the need to assess behavior problems in pediatric patients born with EA in routine care. In addition, these assessments should use both parental and child self-reports to broaden the view of family needs. Parental perceptions of their children's internalizing and externalizing behavior problems differ from those of their children, underscoring that parents of EA patients show greater sensitivity to potentially deviant behavior. In particular, the period following the initial diagnosis, the necessary surgeries, and the reintegration into everyday family life is a delicate phase for parents. The challenges they might experience can impair their empathetic understanding of the sick child's inner world. Likewise, associated anomalies may complicate the ability to resource child-centered perceptions.

Clinicians, using such assessments, may identify vulnerable pediatric patients who could benefit from further assessment and referral. Clinicians should be especially aware that children with EA are at increased risk for internalizing problems, particularly infants and young children, and pediatric patients with associated anomalies. Incorporating routine psychological assessment into pediatric health care can help improve understanding of the burden of illness, examine treatment outcomes, assess the quality of care, and tailor interventions to meet patient and parent needs.

Interventions or psychosocial support should also consider whole families, including pediatric patients, parents, and siblings, especially in the early years after the initial diagnosis and in the presence of associated anomalies.

Involving the whole family can help develop appropriate and functional coping strategies. From our point of view, it is necessary to address parental needs and concerns as well in order to provide the best possible holistic development in the family system. The family is the basis for the children's successful development, especially for children with special health care needs.

## Data Availability Statement

The data analyzed in this study is subject to the following licenses/restrictions: The data set is available for request. Requests to access these datasets should be directed to s.witt@uke.de.

## Ethics Statement

The studies involving human participants were reviewed and approved by Hannover Medical School. Written informed consent to participate in this study was provided by the participants' legal guardian/next of kin.

## Author Contributions

SW, MD-B, JD, and JQ developed the study concept and the design. MD-B developed the study materials. SW, JQ, and JD acquired the data. SW and JQ analyzed and interpreted the data. SW wrote the first draft of the manuscript. MD-B, JD, and JQ revised the first draft critically for important intellectual content. All authors have revised the subsequent drafts critically, approved the final manuscript to be published, and agreed to be accountable for all aspects of the work.

## Conflict of Interest

The authors declare that the research was conducted in the absence of any commercial or financial relationships that could be construed as a potential conflict of interest.

## Publisher's Note

All claims expressed in this article are solely those of the authors and do not necessarily represent those of their affiliated organizations, or those of the publisher, the editors and the reviewers. Any product that may be evaluated in this article, or claim that may be made by its manufacturer, is not guaranteed or endorsed by the publisher.
